# When dinosaurs hear like barn owls: pitfalls and caveats in assessing hearing in dinosaurs

**DOI:** 10.1098/rsbl.2024.0680

**Published:** 2025-05-07

**Authors:** Geoffrey A. Manley, Christine Köppl

**Affiliations:** ^1^ Department of Neuroscience, and Cluster of Excellence "Hearing4all", and Research Centre Neurosensory Science, Carl von Ossietzky Universität Oldenburg, Oldenburg, Niedersachsen, Germany

**Keywords:** dinosaurs, hearing, auditory, cochlea, evolution, birds

## Abstract

Computer tomographic scanning is now a standard technique for studying the internal features of fossil structures. This enables comparisons with related modern species and speculation concerning function and even behaviour. We express here a concern that inferences about dinosaur hearing and further implications about, e.g. communication or hunting skills, are sometimes stretched beyond what can reasonably be gleaned from fossil data. We summarize current knowledge about structure–function relationships in the avian auditory inner ear and provide guidance for evidence-based inference of hearing capabilities from bony features. In particular, we point out limitations and caveats regarding inferences that are based on one isolated feature alone, typically cochlear length. As an example illustrating some of these pitfalls, we use a recent analysis (Choiniere *et al*. 2021 *Science* **372**, 610–613 (doi:10.1126/science.abe7941)) that concluded that *Shuvuuia deserti,* a theropod dinosaur, showed pronounced sensory specializations, including ‘specialized hearing acuity, rivalling that of today’s barn owl’. We re-analysed the skeletal features of *Shuvuuia’s* inner ear and argue that the analogy between hearing in *Shuvuuia* and the extant barn owl was based on an ill-chosen metric in assessing the relative length of the cochlear duct and a questionable assumption concerning inner-ear structure.

## 1. Introduction

Fossil specimens provide intriguing glimpses into worlds long past, and the morphological detail available from state-of-the-art imaging techniques is ever-improving [1]. The challenge then lies in the interpretation of such morphological data regarding function and the general ecology of the fossil species. This must necessarily be based on current knowledge of structure–function relationships in extant species.

Dinosaurs have long carried a particular fascination for researchers and the public alike. The sensory capabilities of fossil species may provide important clues to their lifestyle and behaviour, and basic data on the size of sensory organs have been used to infer their relative importance in extinct species (e.g. [2–4]). Moreover, the emerging field of palaeoneurology enables increasingly detailed inferences from brain endocasts [5–7]. For hearing, the main indicators typically derived from fossil specimens are the length and shape of the endosseous cochlear duct (ECD) [8,9]. Using functional data about the inner ear of birds, the only surviving dinosaur clade, inferences are made about the hearing ranges of different species of dinosaurs.

We express here a concern that inferences about dinosaur hearing and further implications, e.g. on communication or hunting skills, are sometimes extrapolated beyond what can currently be gleaned from fossil data. We summarize relevant knowledge about structure–function relationships in the avian auditory inner ear and provide evidence-based recommendations for functional interpretations from limited morphological data. In particular, we point out limitations and caveats regarding inferences that are based on one isolated feature alone, such as cochlear length. Furthermore, we caution interpretations of unusual morphological features as indicating hearing specializations. Similar summaries for the mammalian cochlea can be found in [10].

We use two species as illustrative examples for potential pitfalls: the extant barn owl, *Tyto alba*, a nocturnal predator and known auditory specialist among birds [11], and *Shuvuuia deserti,* a fossil theropod dinosaur that was recently claimed to show analogous hearing specializations to the barn owl. Choiniere *et al*. [1] reported an analysis of theropod dinosaurs and concluded that the Alvarezsauroidea, a group of nocturnal predators, showed pronounced sensory specializations of extremely low-light vision and profound hearing acuity. One species in particular stood out: ‘The Late Cretaceous alvarezsauroid *Shuvuuia deserti* had even further specialized hearing acuity, rivaling that of today’s barn owl’ [1, p. 610].

## 2. What does cochlear-duct length mean for auditory function?

First, we summarize three important caveats about deducing auditory function, such as the hearing range and frequency of best hearing, on the basis of ECD length alone.

In vertebrates, the inner ear houses the organs of both hearing and balance. In birds and other dinosaurs, the auditory organ (basilar papilla) lies within a tube-like cochlea that at its apex also harbours a smaller vestibular organ, the lagenar macula. The basilar papilla of birds has been the subject of several reviews (e.g. [12–14]). In most extant bird species, the basilar papilla is between a few and 6 mm in length [12]. Only highly specialized owls have much longer papillae (barn owl: 11 mm). Frequencies are arranged tonotopically, with the lowest frequency at the apex: the wider the apical papilla, the lower the frequency. Larger birds tend to have longer but also wider papillae [12], and both these dimensions influence the audible frequency range: a general inverse correlation exists between body size or basilar-papilla length and the frequency of most sensitive hearing, such that larger species with longer basilar papillae tend to show their best hearing at lower frequencies than smaller species with shorter, narrower basilar papillae [8,13]. Thus, when comparing the audible range and cochlear dimensions in modern species, the organ dimensions in extinct species, including dinosaurs, permit some predictions about audition.

### (a) Caveat no.1: the length of the (endosseous) cochlear duct is not equal to the length of the sensory basilar papilla

As in all non-mammals, the lagenar macula is an organ covered by otoliths and, in the only functional study in land vertebrates (chickens), was found to be non-auditory [15]. On average, the space occupied by the lagenar macula was found by Gleich *et al*. [8] to reduce the length of the auditory basilar papilla to 2/3 of that of the entire cochlear duct. However, no absolute lengths were provided, and the derivation of the proportion was not detailed. Our own preliminary sample of lagenar size in three extant bird species (see electronic supplementary material) indicates that 1/3 was for some species an overly generous estimate. Furthermore, lagenar length varied much less across species than the known basilar-papilla lengths. In particular, our measurements in the barn owl indicate that its lagena occupies less than 10% of the ECD length, suggesting that elongation of the hearing organ was not accompanied by a proportional enlargement of the lagena. We thus believe that the size relationship between basilar papilla and lagena needs a more comprehensive study and that a fixed proportion between both does not hold across the full range of ECD lengths. *Shuvvuia’s* ECD was 5.4 [16] to 6.5 [1] mm long, leading to a maximum range estimate of somewhere between 3.6 and 5.2 mm for its basilar papilla. This length is similar to that of a modern chicken, a bird with a similar body size to *Shuvuuia*, and less than half of the papillar length in the barn owl [12].

### (b) Caveat no. 2: frequency of best hearing and upper hearing limit may be cautiously extrapolated from extant data

Gleich *et al*. [8] provided an equation that may be used for estimating the frequency of best hearing in archosaurs, but note that they used basilar-papilla length, not ECD. Thus, for fossil species, this requires an assumption about the proportion of the ECD occupied by the sensory basilar papilla (see §2*a*). As an example, for *Shuvuuia*, with an assumed basilar-papilla length of between 3.6 and 5.2 mm (see above), an estimate of best hearing between 1.6 and 2.3 kHz results.

For extant birds, the frequency of best hearing is, in turn, tightly related to the upper-frequency limit of sensitive hearing [8,13], such that it can be reliably estimated as 1.1 octaves above the frequency of best (most sensitive) hearing [13]. Again, as an example, this would result in a range of 3.4–5 kHz as the high-frequency limit of hearing in *Shuvuuia*, as compared to >10 kHz in the barn owl [14]. The lower frequency limit of sensitive hearing also shows an inverse correlation with basilar-papilla length, but is much more variable [13]. This is consistent with known mechanisms of frequency tuning: in birds, the mechanical frequency responses of sensory hair cells become less dominant at low frequencies, where hair cells are also electrically tuned (for a review, see [17]). The membrane ion channel constellation in each hair cell differs systematically along the papilla and cannot be assessed from anatomical data. Unfortunately, in the absence of bony supports for the organ, the width of the papilla, which influences frequency responses, cannot be determined in fossil dinosaur skulls. This factor, which was largely ignored by Gleich *et al.* [8] and Walsh *et al.* [9] makes predictions of the audible range of large fossil species more speculative. We, therefore, recommend refraining from estimating the lower frequency limit of hearing from ECD or basilar-papilla length.

As a final word of caution, for extinct species of great body mass, all estimates represent extrapolations far beyond the range of data from extant species (body mass <60 kg) [8].

### (c) Caveat no. 3: no informed guesses are possible about other aspects of hearing such as sensitivity or frequency resolution

Many features, including hair-cell anatomy and ion channels, together determine the frequencies processed along the avian basilar papilla. The relative importance of those factors differs along the tonotopic gradient, such that the space constant (the length of the basilar papilla devoted to one frequency octave) varies not only between species but also within one species’ basilar papilla. For extant bird species for which this space constant is known, a correlation was shown with behavioural measures of frequency discrimination [13]. We believe that such correlations cannot be extrapolated to fossil data. Although a generic equation exists for predicting the frequency map for a given basilar-papilla length, this requires knowledge about the limits of the species’ hearing range [12], which, if only ECD length is available, in turn requires several assumptions in itself (see §2*b*). Furthermore, species with outlying ECD length, such as the barn owl, do not conform to the general trend [13]: although the barn owl shows an extreme space constant in the high-frequency regions of its basilar papilla, consistent with its elongation, frequency *selectivity* in the barn owl is not exceptional among birds [18,19].

Regarding the sensitivity of hearing, there is no evidence that thresholds are predicted by ECD length. Once more, the barn owl is an instructive example that underscores caution in trying to do so. Although the barn owl’s—and owls’ generally—best auditory threshold is known to be near −20 dB SPL (roughly 20 dB more sensitive than other birds [18]), this improvement is produced by the feathery facial ruff [20] and not by its unusually long basilar papilla.

## 3. Beyond endosseous cochlear-duct length: what else may be gleaned from fossils?

In this second part, we highlight three more caveats related to identifying cochlear specializations and interpreting unusual features in fossil material.

### (a) Caveat no. 4: how to assess relative length of the cochlear duct: relative to what?

A first indicator for auditory specializations may be an exceptionally large inner ear. To identify disproportionately long basilar papillae, studies relating ECD to body metrics have used body mass [13] or basicranial length [9]. Both metrics were verified with data from extant species and reliably identified the barn owl, a known auditory specialist, as an outlier with an exceptionally long cochlear duct. As the following example of *Shuvuuia* illustrates, there are serious pitfalls in choosing an appropriate body metric for this normalization.

#### (i) Case pitfall: a particular choice of body metric led to an erroneous claim

Choiniere *et al.* [1] claimed a disproportionately long ECD in *Shuvuuia* and pointed out a supposed analogy to the barn owl, with both species falling clearly above an average phylogenetic scaling rule between skull size and ECD length. However, this analogy depends critically—and we believe erroneously—on the choice of the relative metric. Choiniere *et al.* [1] chose ECD length relative to braincase height. Importantly, the relative metric thus derived confounds inner-ear evolution with brain evolution. Extant birds have relatively large brains compared to their dinosaurian ancestors [21]. As we detail below, *Shuvuuia’s* ECD length relative to braincase height appears to be in the same league as the barn owl’s, not because of *Shuvuuia’s* unusual ECD length but because of its small brain.

**Figure 1.**
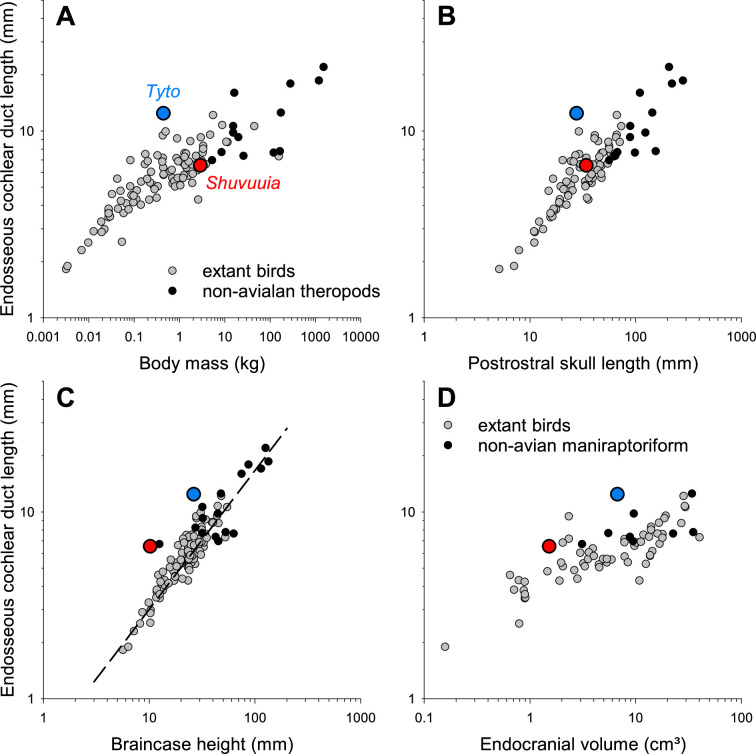
ECD length as a function of four different body metrics: body mass (A), skull postrostral length (B) and braincase height (C) show data for a sample of 97 extant birds (grey symbols) and 17 non-avialan theropods (black symbols), re-plotted from Choiniere *et al*.’s [1] electronic supplementary material (file ‘Lagena length data 24 January 2021.csv’); panel (C) also includes the regression line of their fig. 3A. For data sources on endocranial volume (D), see legend of figure 2. The blue symbol highlights barn owl data, the red, *Shuvuuia*.

**Figure 2.**
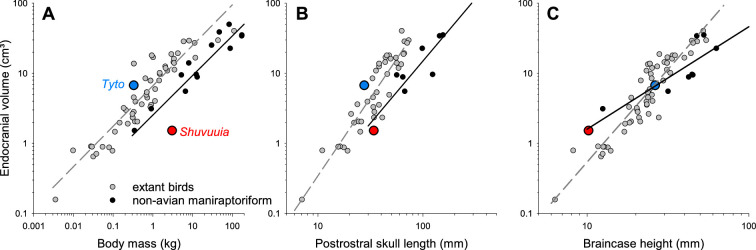
Endocranial volume as a function of three different body metrics: body mass (A), skull postrostral length (B) and braincase height (C). Data were combined from electronic supplementary material in [1] (file ‘Lagena length data 24 January 2021.csv’), in [21] (file ‘Ksepka_DataS1_mmc3.xlsx’) and in [5] (file ‘ar25459-sup−0002-files2.xlsx’ and their table 1), to produce a total sample of 61 extant birds (grey symbols), and 13 non-avian theropods (black symbols). Note we restricted this sample to maniraptoriform theropods because their endocranial volumes are the least controversial and require no further correction factors [5]. Power regressions (dashed grey and solid black lines) were added as visual aids emphasizing trends.

Using the data provided in their electronic supplementary material, we re-plotted the data of Choiniere *et al*. ([1], their fig. 3A) in figure 1C and compared this to equivalent plots using body mass, or skull postrostral length (which is similar to basicranial length), as used in previous studies relating ECD to body metrics [9,13]. It is obvious that *Shuvuuia* only and specifically stands out when relating ECD to braincase height (figure 1C), but not when relating it to either body mass (figure 1A) or skull postrostral length (figure 1B). In contrast, the barn owl clearly stands out in all of these measures (figure 1A–C). So, what is different regarding braincase height? Braincase height and brain volume show a particularly tight correlation and more uniform regression across taxa (figure 2C). In contrast, body mass and skull postrostral length also correlate to brain volume, but birds and their closest theropod relatives (non-avian maniraptoriform theropods) separate more clearly into different populations (figure 2A,B).

Such quantitative data are also consistent with the observation that with increasing volume, brain shape grew disproportionately in the dorsoventral dimension, while remaining compact in the rostrocaudal axis [22]. Thus, relating ECD to braincase height and using the residuals to indicate outliers—as used by Choiniere *et al*. [1]—cannot distinguish between an unusual ECD length and a relatively small endocranial volume. Large datasets on endocranial volume [21] suggest that non-avialan dinosaurs had smaller brains when compared to extant bird species. Indeed, *Shuvuuia* in particular stands out with a relatively small brain even among its closest relatives, the maniraptoriform theropods ([5] and figure 2A). We thus suggest that the major determinant for *Shuvuuia’s* outlying residual value in the analysis by Choinere *et al*. [1] is its small, flat brain and not a long cochlear duct. Comparing figure 1A,B to figure 1C,D suggests that the same issue will apply to any brain metric (here, braincase height and endocranial volume), while *Shuvuuia’s* ECD is not unusual in relation to other body metrics (here, body mass and postrostral skull length).

### (b) Caveat no. 5: beware of species that deviate from general allometric scaling rules

What if future evidence for such specialization is truly found for a fossil dinosaur? Exciting as such findings may be, we advise caution in claiming specific hearing capabilities, for the reasons detailed here.

If body size is controlled for in extant species, correlations of ECD with overall hearing range and centre frequency remain, however, more scatter [9]. This may partly be due to not considering the basilar-papillar width. Species with relatively long ECD tend to have wider hearing ranges and higher centre frequencies. Equations for estimating these hearing parameters were provided by [9], but note that the metric the authors used for normalizing ECD was basicranial length, not body mass. We urge caution in the interpretation of such hearing estimates, because of the scatter in the underlying data from extant species. Most importantly, data from species with increasingly deviant ECD (or basilar-papilla) length, such as the barn owl, clearly do not conform to any of the typical hearing correlations [8,13] and still show large residuals even after controlling for allometric scaling [9]. While such clear deviations may indicate specialized hearing capabilities, the nature of the specialization cannot be inferred from ECD or basilar-papilla length.

Interestingly, Choiniere *et al*. [1] did not claim specific hearing abilities for *Shuvuuia,* although their repeated analogy to the barn owl may be understood as such. However, another large comparative analysis of dinosaur inner ears, including *Shuvuuia*, used Walsh *et al*.’s [9] equations to estimate hearing ranges [16]. *Shuvvuia’s* hearing did not stand out, whereas the barn owl’s once more did.

### (c) Caveat no. 6: care is needed in the interpretation of novel features in extant species before extrapolating to fossils

#### (i) Case pitfall: there are no bony laminae, analogous to the mammalian cochlea, in the avian cochlear duct

As a further unusual feature claimed to be shared by the barn owl and *Shuvuuia*, Choiniere *et al*. [1] identified in both species ‘… two well-defined laminae that extend along the length of the endosseous cochlear duct, medially (primary bony lamina) and laterally (secondary bony lamina). Similar laminae in mammals are attachment sites for the basilar membrane, which supports the basilar papilla’. We believe that this interpretation confuses two kinds of structure that only superficially resemble each other. In mammals, both primary and secondary bony laminae are found in cochlear regions whose frequency responses exceed 10 kHz [23]. In the course of their evolutionary history, therian mammalian cochleae were invaded by bone, and bony supports from the neural (modiolar) and—in high-frequency regions—abneural sides penetrate deeply up to the basilar membrane. Very thin bony platforms (hence the term laminae) reach the edges of the basilar membrane, and the ‘laminar gap’ between them approximates its width [24]. Such a bony encroachment has never been observed in reptiles or birds.

**Figure 3.**
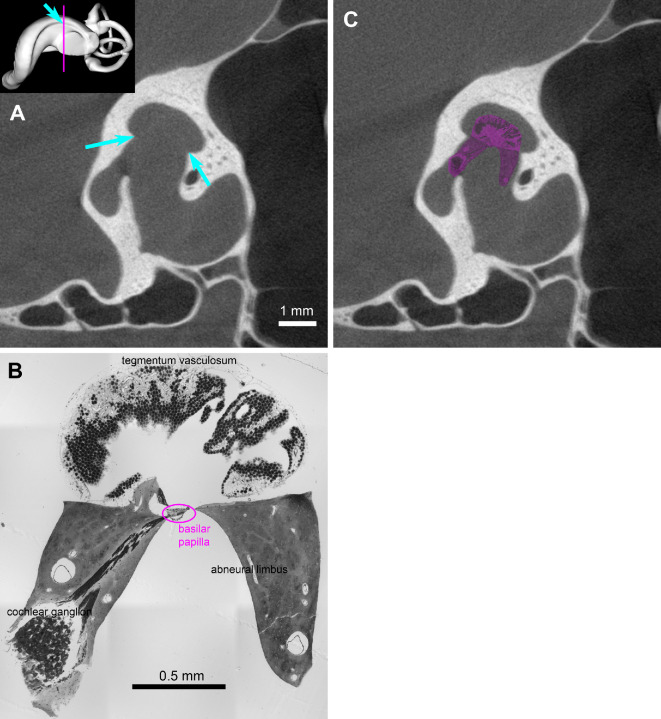
Soft-tissue structure of the auditory inner ear, in relation to the surrounding bone, in the barn owl *T. alba*. (A) The inset on the top left shows a 3D rendering of the left labyrinth. The cyan arrow in the inset points to the ‘secondary bony lamina’ identified by [1]; the magenta line indicates the approximate position of cross-section represented by the micro-CT scan layer shown in the main panel. Cyan arrows in the main panel point to the two bony indentations identified as primary and secondary laminae by [1]. Scale bar corresponds to 1 mm. (B) Histological cross-section of the soft tissue inside a barn owl cochlear duct (for Methods, see electronic supplementary materiall), at approximately the same level as the micro-CT layer shown in (A). Major structures are identified. Note in particular the magenta oval encircling the basilar papilla situated on the basilar membrane. Scale bar corresponds to 0.5 mm. (C) The same micro-CT layer as in (A), shown together with the soft-tissue section from (B), now coloured in magenta and adjusted to the same magnification. Roger Benson provided access to the *Tyto* labyrinth reconstruction used in (A), originally appearing in [1], the collection of which was funded by the European Research Council (ERC) starting grant TEMPO (ERC-2015-STG-677774) to Roger Benson. Jeffrey Zeyl *et al*. provided access to the micro-CT scan of a *Tyto* skull used in (A) and (C) [25]. The files were downloaded from https://www.MorphoSource.org, Duke University.

In *Shuvuuia*, the ‘secondary bony lamina’, shown in figure 2 of Choiniere *et al*. [1], is merely a broad indentation, very unlike the thin laminae of mammals. In the barn owl, to which they compare *Shuvuuia*, the space between the bony indentations is very wide and comfortably accommodates the entire endosseous structure (figure 3). The bone does not approach anywhere near the basilar membrane with its sensory basilar papilla. The tissue that supports the edges of the basilar membrane in the barn owl—as in all other birds and in modern reptiles—is cartilaginous (neural and abneural limbus in figure 3B). The bony indentations discernible in endosseous casts of barn owls and *Shuvuuia* are thus not comparable to the bony laminae of mammalian cochleae. We highly recommend seeking input from auditory experts when interpreting any novel or unusual features in fossil inner ears.

## 4. Conclusions

Inferring the sensory capabilities of extinct species is a fascinating exercise and potentially insightful into their ecology. However, we believe that the desire to learn about the auditory world of fossil dinosaurs has occasionally led to claims that were not sufficiently evidence-based. We thus argue for more caution when assessing auditory capabilities in fossil species and provide guidelines for doing so in avialan and non-avialan relatives.

Using the example of the non-avialan theropod *S. deserti*, we illustrate specific pitfalls and conclude that inferences about exceptional hearing abilities were misled by a particular choice of relative metric that appeared to show a disproportionally long cochlear duct. Instead, we suggest that the outlying metric represents a relatively flat brain, unrelated to anything auditory. Furthermore, we conclude that the identification of bony laminae in cochlear endocasts of both *Shuvuuia* and the modern barn owl was flawed. In the barn owl, these structures do not penetrate close to the edges of the basilar membrane and are thus not analogous to structures in mammalian cochleae. Together, these invalidate the claim that hearing in *Shuvuuia* was specialized or unusual.

## Data Availability

Data are fully detailed in the electronic supplementary material. Supplementary material is available online [26].
